# Genome‐wide profiles of DNA damage represent highly accurate predictors of mammalian age

**DOI:** 10.1111/acel.14122

**Published:** 2024-02-23

**Authors:** Huifen Cao, Bolin Deng, Tianrong Song, Jiabian Lian, Lu Xia, Xiaojing Chu, Yufei Zhang, Fujian Yang, Chunlian Wang, Ye Cai, Yong Diao, Philipp Kapranov

**Affiliations:** ^1^ Institute of Genomics, School of Medicine Huaqiao University Xiamen China; ^2^ Department of Clinical Laboratory the First Affiliated Hospital of Xiamen University Xiamen China; ^3^ Xiamen Cell Therapy Research Center the First Affiliated Hospital of Xiamen University Xiamen China; ^4^ Changping Laboratory Beijing China; ^5^ State Key Laboratory of Cellular Stress Biology, School of Life Sciences Xiamen University Xiamen China

**Keywords:** abasic site, aging, biomarkers, breakome, machine learning, single‐strand DNA break, SSiNGLe, SSiNGLe‐AP

## Abstract

The identification of novel age‐related biomarkers represents an area of intense research interest. Despite multiple studies associating DNA damage with aging, there is a glaring paucity of DNA damage‐based biomarkers of age, mainly due to the lack of precise methods for genome‐wide surveys of different types of DNA damage. Recently, we developed two techniques for genome‐wide mapping of the most prevalent types of DNA damage, single‐strand breaks and abasic sites, with nucleotide‐level resolution. Herein, we explored the potential of genomic patterns of DNA damage identified by these methods as a source of novel age‐related biomarkers using mice as a model system. Strikingly, we found that models based on genomic patterns of either DNA lesion could accurately predict age with higher precision than the commonly used transcriptome analysis. Interestingly, the informative patterns were limited to relatively few genes and the DNA damage levels were positively or negatively correlated with age. These findings show that previously unexplored high‐resolution genomic patterns of DNA damage contain useful information that can contribute significantly to both practical applications and basic science.

## Graphical abstract

This study explored the potential of high‐resolution genomic patterns of single‐strand breaks and abasic sites, which were identified by methods recently developed by our group, as a source of novel age‐related biomarkers in mice. We found that the amount of either DNA lesion in relatively few genes could be used to accurately predict age, and the DNA damage levels in these genes were either positively or negatively correlated with age. These results suggest that previously unexplored high‐resolution genomic patterns of DNA damage contain important information that can contribute significantly to both practical applications and basic science.

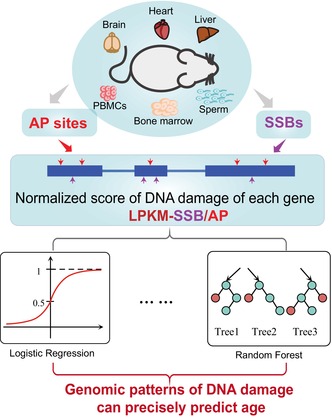

**Abbreviations**
ANOVA: analysis of variance
AP site: abasic site
AUC: area under the curve
BER: base excision repair
DDR: DNA damage response
GO: gene ontology
LASSO: least absolute shrinkage and selection operator
LGBM Classifier: light gradient boosting machine classifier
LPKM: lesions per kb per million
MAE: mean absolute error
RNA‐seq: RNA sequencing
SSB: single‐strand DNA break
SSiNGLe: single‐strand break mapping at nucleotide genome level
TPM: transcripts per million

## 1 INTRODUCTION

Aging poses a significant risk factor for chronic diseases and functional decline (Atella et al., 2019; Hou et al., 2019; Kennedy et al., 2014; Niccoli & Partridge, 2012). As the proportion of the global population aged 65 years or older continuously increases (UN, 2019a, 2019b), the impact of aging on humankind has become more pronounced, calling for a deeper understanding of the root causes of aging and development of potential approaches to counter the effects of this phenomenon. The identification of age‐related molecular changes can be beneficial for both; they could uncover the molecular processes responsible for aging and give rise to age‐related biomarkers that could be used to more precisely estimate the true biological age of an individual (Bao et al., 2023; Xia et al., 2017). Age estimation could then lead to potential recommendations for lifestyle changes or perhaps even therapeutic interventions with aging‐slowing drugs (Kennedy et al., 2014).

Multiple omics‐based studies resulted in the detection of a multitude of aging‐related changes that take place in transcriptome (Angelidis et al., 2019; Jia et al., 2018; Peters et al., 2015; Qi et al., 2021), epigenome (Horvath, 2013; Levine et al., 2018; Lu et al., 2019), proteome (Angelidis et al., 2019; Lehallier et al., 2020), metabolome F (Robinson et al., 2020; van den Akker et al., 2020), gut microbiome (Chen et al., 2022; Galkin et al., 2020) and other systems at the levels of cells, organs or whole organisms in humans and other species (reviewed in Bao et al., 2023). The wealth of identified age‐related events has led to the development of several databases, such as the Human Aging Genomic Resources (Tacutu et al., 2018), Aging Atlas (Aging Atlas, 2021), and MetaboAge DB (Bucaciuc Mracica et al., 2020), which are dedicated to cataloging this information. Naturally, the availability of measurable age‐related features, patterns, and biomarkers of aging has been used to generate multiple models—clocks‐that can predict the chronological or biological age of an individual (Bao et al., 2023). Biological age represents the health status of an individual, which might differ from chronological age, and thus represents a highly clinically relevant metric (Andrews et al., 2017; Nie et al., 2022). Impressive progress in the development of biological clocks has resulted in several clinically significant models (Li et al., 2020). However, current biological clocks are not perfect, as indicated by at least two major issues. First, the concordance between various biological clocks ranges from low to moderate (Belsky et al., 2018; Li et al., 2020), raising concerns regarding the accuracy and sensitivity of these models. Second, the hazard ratios of even the best‐aging clocks are fairly modest (Li et al., 2020). These issues are not surprising considering the complexity of the aging process, and the likely solution to these problems lies in the development of new types of age‐related biomarkers (Bao et al., 2023).

In this regard, there is a strong gap in age biomarkers based on genome‐wide profiles of DNA damage, which is striking considering the central role played by DNA damage in multiple theories of aging (Campisi, 2013; Chen et al., 2020; Hoeijmakers, 2009; Maynard et al., 2015). The main reason for this gap is the lack of genome‐wide, high‐resolution techniques for profiling different types of DNA lesions. To address this issue, our group recently developed two methods, SSiNGLe and SSiNGLe‐AP, which, for the first time, allowed for high‐resolution detection of two very common types of DNA damage, single‐strand DNA breaks (SSBs) and abasic (AP) sites, in complex mammalian genomes (Cai et al., 2022; Cao et al., 2019). Using these approaches, we identified associations between aging and genome‐wide patterns of DNA damage; however, these patterns were inconsistent across different tissues (Cai et al., 2022).

In the current study, we further explored the potential of high‐resolution genome‐wide profiles of SSBs and AP sites as age‐related biomarkers. We estimated the levels of each of the two types of DNA lesions for each gene in several tissues of mice of various ages. Using multiple machine‐learning methods, we used this information to identify age‐associated genes and develop various aging clocks based on these genes. We found that models based on DNA damage‐based metrics could achieve highly accurate age predictions, with area under the curve (AUC) values as high as ~1, which were significantly higher than those obtained using transcriptome analysis based on RNA extracted from the same samples. These results suggest that genome‐wide patterns of DNA damage represent a novel source of biomarkers for predicting biological age across diverse tissues and potentially for other biological conditions. We found that, depending on the gene, the levels of SSBs or AP sites could either positively or negatively correlate with age, which is contrary to the general assumption that the levels of all types of DNA damage universally increase with age. Overall, our study highlights that the nonrandomness of genomic patterns of DNA damage depends on the biological state and underscores the importance of DNA damage as a fundamental component of the aging process.

## 2 MATERIALS AND METHODS

### 2.1 Biological material

Animal maintenance conditions, tissue harvesting, and DNA isolation have been described in our previous work (Cai et al., 2022) and are briefly listed below. All mice were housed under pathogen‐free conditions with a 12‐h light/dark cycle, an ambient temperature of 22–25°C, humidity from 40% to 60%, and ad libitum access to food and water at the Xiamen University Laboratory Animal Center. Motile spermatozoa were isolated from the mouse epididymal tail by collecting cells floating in the upper layer of RPMI 1640 medium (Thermo Fisher Scientific, C11875500BT). PBMCs were isolated from blood using Ficoll Paque Plus (GE Healthcare, 17–1440‐03). Red blood cells in the bone marrow were removed using Red Blood Cell Lysis Buffer (Beyotime, C3702‐500 mL). After harvesting, the tissues were immediately placed in liquid nitrogen. Whole organs were cut into small pieces and ground into a powder using a tissue homogenizer in the presence of liquid nitrogen. Genomic DNA from sperm cells was extracted using a Sperm DNA Purification Kit (Simgen, 4,202,050), whereas genomic DNA from all other tissues was isolated using a TIANamp Genomic DNA Kit (Tiangen, DP304‐03) with RNase A (Tiangen, RT405‐12) without proteinase K treatment.

### 2.2 LPKM‐SSB/AP and TPM calculation

The DNA damage level (SSB or AP sites) of gene *i* in each sample was calculated using the following formula:

$$\text{LPKM}-\mathrm{SSB}/\mathrm{AP}=\frac{\mathrm{Ri}*10^{10}}{\text{Total number of positions of SSBs or}\;\mathrm{AP}\;\text{sites}}$$

where Ri is the number of positions of SSB or AP sites located in the exon(s) or the entire region of gene *i*. TPM was calculated using the RSEM package (Li & Dewey, 2011).

### 2.3 ANOVA

To select the age‐ or tissue‐related genes, the analysis variance as implemented by the function of “f_classify” in the sklearn package was used to calculate the *F*‐score (*F*) for gene *i* and each of the three metrics in the sample *j* as illustrated below for LPKM‐SSB and age.

$$F=\frac{\mathrm{MSB}}{\mathrm{MSE}}.$$

MSB and MSE represent the variance between different age groups and within a group, respectively, and were calculated as follows:

$\mathrm{MSB}=\frac{\sum_{j=1}^{c}n_{j}{\left(\bar{x_{j}}-\bar{\bar{x}}\right)}^{2}}{c-1}$, in which, $\sum_{j=1}^{c}n_{j}{\left(\bar{x_{j}}-\bar{\bar{x}}\right)}^{2}$ is the horizontal sum of squares of the level of LPKM‐SSBs of the gene across the different age groups the “*c*−1” term denotes the degrees of freedom for the age groups; $\bar{x}=\frac{\sum_{j=1}^{c}\sum_{i=1}^{n_{j}}x_{\mathrm{ij}}}{n_{r}}$, represents the mean value of the SSB levels of the gene in all samples; *n*_*r*_ represents the sample size.

$\mathrm{MSE}=\frac{\mathrm{SSE}}{n_{r}-c}$, where $\mathrm{SSE}=\sum_{j=1}^{c}\sum_{i=1}^{n_{j}}\left(x_{\mathrm{ij}}-\bar{{x_{j}}^{2}}\right)$, represents the sum of squares of the error term, $n_{r}-c$ is the degree of freedom for the SSE.

The genes were sorted by *F*‐score, with the gene having the highest *F*‐score assigned a rank of 1, and the top 500 genes for each metric were then selected.

### 2.4 Machine‐learning algorithms

The 10 different methods used for age and tissue prediction in this work were chosen mainly because of their wide application in the machine‐learning field and are briefly summarized below.

1. Elastic Net: a statistical technique that combines L1 and L2 regularization in linear regression and is used for feature selection and sparse modeling.
2. Random Forest Classifier: an ensemble‐based machine‐learning model consisting of multiple decision trees that perform classification by voting or averaging predictions.
3. Decision Tree Regressor: a nonparametric supervised learning algorithm used for regression problems that predict the target variable by constructing a decision tree.
4. Decision Tree Classifier: a nonparametric supervised learning algorithm is used for classification problems that predict class labels by constructing a decision tree.
5. AdaBoost Classifier: an ensemble‐based classification algorithm that iteratively trains multiple weak classifiers and combines them into a single strong classifier.
6. Logistic Regression: A generalized linear model is used for binary or multiclass classification problems that predict class probabilities by combining a linear function with a logistic function.
7. Gaussian NB: a Naive Bayes classifier based on the assumption of feature independence and a Gaussian distribution.
8. LGBM Classifier: a light gradient boosting machine classifier based on a gradient boosting framework that uses histogram‐based algorithms for fast and efficient training.
9. XGB Classifier: an ensemble learning algorithm based on gradient boosting trees that improves the performance and robustness of the model by adding regularization techniques to the gradient boosting.
10. LASSO: a linear regression model incorporating L1 regularization for feature selection and sparse modeling.

All these methods were applied using Python version 3.10.9. The LGBM and XGB classifiers were executed using the packages lightgbm (v3.3.5) and xgboost (v1.7.4), while the rest of the eight methods were executed using scikit‐learn (v1.2.2). The AUC, F1, MAE, and Spearman correlation values were calculated using scikit‐learn (v1.2.2). The R package ClusterProfiler (Wu et al., 2021) was used for GO analysis, and the package ggplot2 was used to plot the figures.

## 3 RESULTS

### 3.1 Genome‐wide profiles of DNA damage as potential sources of age‐related biomarkers

In this study, we used genome‐wide profiles of SSBs, AP sites, and RNA from six tissues (brain, bone marrow, heart, liver, peripheral blood mononuclear cells (PBMCs) and sperm) of mice from four chronological age groups (3, 12, 19, and 22 months), which have been previously published by our group (Cai et al., 2022), to which we have added new data of the same type and tissues from 24‐month‐old mice generated in this study. Each of the 30 time‐point‐tissue combinations (five age groups × six tissues) was represented by three C57BL/6J mice, with the exception of the 24‐month age group, where each tissue was harvested from two animals, resulting in a total of 83 samples (one brain sample was excluded after quality control). The DNA used for the genome‐wide profiling of SSBs and AP sites and the RNA used for transcriptome analysis were obtained from the same tissue samples.

**FIGURE 1:**
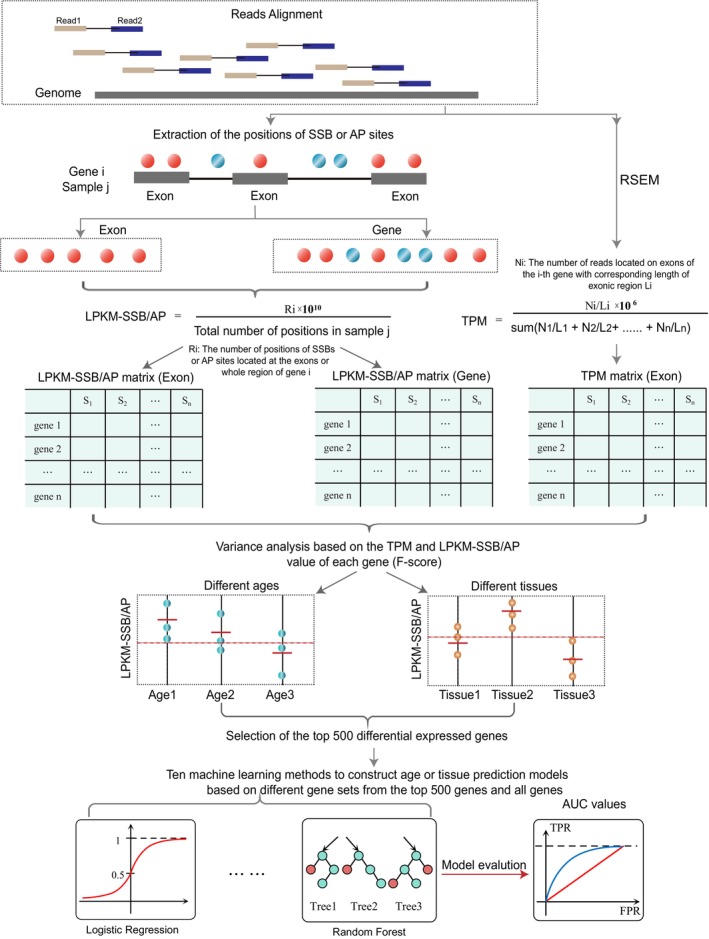
The process of constructing the age and tissue prediction models. The single‐nucleotide positions of SSBs and AP sites were used to calculate respectively the LPKM‐SSB and LPKM‐AP metrics for each gene in each sample, while the entire RNA‐seq reads were used to calculate corresponding TPM values. The three metrics were then used to calculate the *F*‐scores for each gene, which were then used to select the top 500 genes that differ the most across the age groups or the tissue types. Multiple machine‐learning methods were then employed to construct the models to predict age or tissue type, and AUC (and the other parameters) were used to evaluate the performance of each model, metric, and gene set.

First, we calculated the expression and DNA damage levels of each annotated mouse gene by counting the RNA‐seq reads or nucleotide‐level DNA lesions mapped to the corresponding exons (Figure 1). The rationale was to ensure that the comparison between DNA damage‐ and transcriptome‐based predictors was based on the same genomic elements. For transcriptome data, we calculated gene expression values based on the transcripts per million (TPM) metric, which is commonly used to analyze RNA‐seq data (Wagner et al., 2012; Zhao et al., 2020). However, because such a metric would not be suitable for DNA damage‐based analysis, we determined the DNA damage levels for each gene using lesions per kb per million (LPKM)‐SSB and LPKM‐AP metrics calculated using a formula similar to the reads per kb per million metric (RPKM): the total number of SSBs or AP sites found on either template or non‐template strands in the exons of a gene is normalized to the total length of all the exons of the gene and the library depth (Figure 1, Section 4). As input for all subsequent analyses, we retained 13,481 genes with nonzero TPM values in at least 40% of the samples. To obtain a comparable number of input genes based on the LPKM‐SSB or LPKM‐AP metrics, we retained 13,310 and 10,240 genes that had nonzero LPKM‐SSB or LPKM‐AP in at least 20% of the samples. Such genes will be referred to as “all genes” for each metric in the analyses below.

**FIGURE 2:**
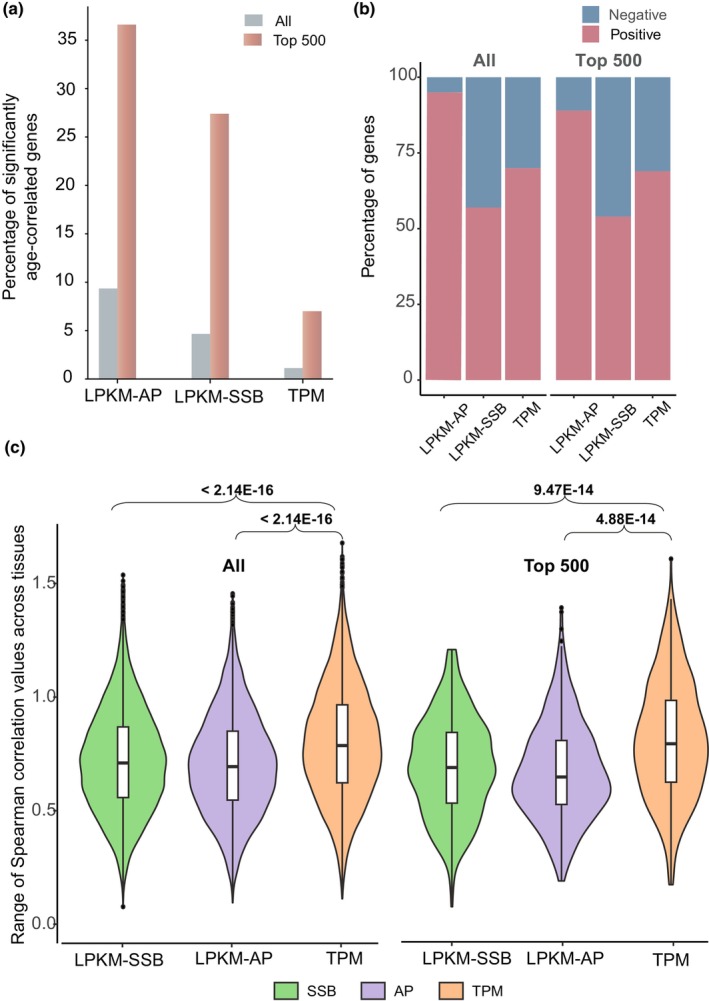
The DNA damage levels in genes correlate more with age than their expression levels. (a) The LPKM‐SSBs and LPKM‐AP metrics detected higher fractions of genes that significantly correlated with age than the TPM metric. The *Y*‐axis represents the fraction of such genes in either the top 500 genes or all genes. (b) The distributions of positively and negatively correlating genes among the genes that had significant correlation with age. (c) For each, gene and metric, the Spearman correlations with age were calculated for each tissue separately and then ranges of the correlations (maximum–minimum) were obtained. The violin plots of the correlation ranges are shown. The *p*‐values were calculated using ANOVA and further adjusted using the Tukey's HSD test.

We then explored whether the DNA damage levels in genes could be used to identify age‐related genes and how well the DNA damage‐based metrics would perform relative to the expression level measurements that have been previously shown to identify age‐related genes in multiple studies (Angelidis et al., 2019; Jia et al., 2018; Peters et al., 2015; Qi et al., 2021). First, we estimated the number of genes significantly (*p* < 0.05) correlated with age using the Spearman correlation coefficient based on the LPKM‐SSB, LPKM‐AP, or TPM metrics. The average Spearman correlation values of the significantly correlated genes were not very large: the average positive correlations were 0.254, 0.262 and 0.251 for the LPKM‐SSB, LPKM‐AP, or TPM metrics, and the corresponding values of negatively correlated genes were − 0.26, −0.254, and − 0.254. However, we found significantly higher fractions of significantly correlated genes using the LPKM‐SSB and LPKM‐AP metrics than using the TPM metric, with corresponding proportions of 4.66% (620 of 13,310), 9.35% (959 of 10,240), and 1.13% (152 of 13,481), respectively (Figure 2a, Tables S1 and S2).

These results suggested that the DNA damage in certain genes can correlate with age at the performance level comparable or even better than that of an established type of a biomarker—the level of expression. However, a linear correlation metric would miss age‐associated genes that exhibit nonlinear relationships with age. Therefore, as the next step, we used a more general approach, analysis of variance (ANOVA), to identify genes which expression or DNA damage levels changed with age (Figure 1, Section 4). Using this analysis, we identified three sets of the top 500 age‐associated genes based on each metric (Table S3). The top 500 genes were more enriched in genes that were significantly correlated with age than all genes: 5.5‐fold (24.7% vs. 4.46%), 3.9‐fold (36.5% vs. 9.35%), and 6.2‐fold (7.0% vs. 1.13%) for the LPKM‐SSB, LPKM‐AP, and TPM metrics, respectively (Figure 2a; Tables S1 and S2). However, we found more age‐correlated genes in the top 500 genes selected based on DNA damage metrics than in those selected based on TPM. Among the top 500 LPKM‐SSB and LPKM‐AP genes, 27.4% (137/500) and 36.3% (183/500), respectively, were significantly correlated with age. These values were 3.9‐ and 5.2‐fold higher than the 7.0% (35/500) of the top 500 genes identified using the RNA‐based TPM metric (Figure 2a; Table S1).

Based on the level of DNA damage, we observed both positive and negative age‐correlated genes (Figure 2b; Table S1). For example, based on all genes, of the 620 and 957 genes that had a significant correlation with age for the LPKM‐SSB and LPKM‐AP, 267 (43%) and 44 (5%) had a negative correlation with age (Figure 2b; Table S1). Thus, the DNA damage levels of these genes decreased with age. Similarly, when limiting the analysis to the top 500 genes, the corresponding numbers of negatively correlated genes were 46% (63/137) and 11% (20/163) for the LPKM‐SSB and LPKM‐AP metrics (Figure 2b; Table S1), respectively.

Next, we investigated the effect of tissue type on the correlation between age and the levels of DNA damage or expression. For each gene, we separately calculated the Spearman correlation values between each of the three metrics and age for each tissue. For each gene, we estimated the range of correlation values across the six tissues. Interestingly, we found that the ranges of correlation coefficients calculated using the TPM metric were significantly larger than those obtained using LPKM‐SSB and LPKM‐AP for the top 500 genes, with adjusted *p*‐values of 9.47E‐14 and 4.88E‐14, respectively (Figure 2c; Table S1). A similar conclusion was reached for all genes (Figure 2c; Table S1). However, no significant differences were observed between the two DNA damage‐based metrics (Figure 2c; Table S1). These results show that the tissue type affected the correlation between age and gene expression levels, which is consistent with previous observations (Shokhirev & Johnson, 2021). However, tissue type had less of an effect on the correlation between age and levels of DNA damage in various genes, providing a potential explanation for why fewer age‐correlated genes were found using the TPM metric across all tissues.

### 3.2 Aging clocks constructed using the DNA damage‐based metrics outperform those based on the transcriptome analysis

The relatively high number of genes that were significantly correlated with age and the relative stability of the correlation across different tissue types, compared with the established transcriptome‐based approach, suggest that the DNA damage‐based metrics developed in this study have strong potential as age predictors. Therefore, as the next step, we applied 10 machine‐learning methods (Logistic Regression, Random Forest Classifier, Naive Bayes, LGBM Classifier, XGBoost Classifier, AdaBoost Classifier, LASSO, Elastic Net, Decision Tree Regression and Decision Tree Classifier) to perform an in‐depth assessment of the properties of DNA damage‐based age predictors and then compared their performance with that of the RNA‐based metric.

**FIGURE 3:**
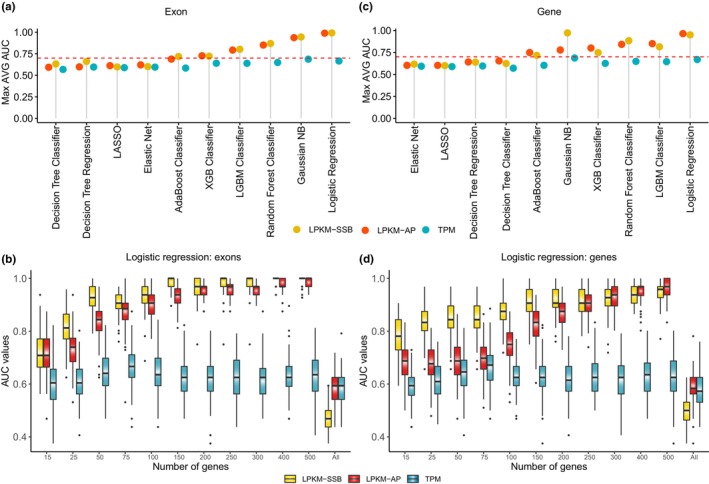
Performance evaluation of the age predicting models generated by the different analytical methods. (a, c) The average AUC values were calculated for each gene set for each method‐metric combination based on the 100 iterations. The maximum of the average AUC values (*Y*‐axes) achieved for each method and metric are shown. (b, d) Boxplots of the AUC values (*Y*‐axes) of the 100 iterations for each gene set (*X*‐axes) and each metric found for the top‐performing method—Logistic Regression. Box plots indicate median (middle line), 25th, 75th percentile (box), and 1.5× interquartile range (whiskers). The DNA damage metrics were calculated using either the exons (a, b) of each gene or whole genes including exons and introns (c, d). The TPM metrics were always calculated using exons only.

For each method and each of the three metrics, we selected 11 gene sets that were used as inputs to construct the age models for the 83 samples. Ten of the 11 gene sets were based on the top 500 genes and generated by first ranking the top 500 genes according to the *F*‐score from ANOVA and then successively expanding the number of genes used to make the models from the top 15 genes to all 500. The 11th gene set represented all genes (described above). For each gene set obtained by each metric and each method, we randomly selected 80% (66/83) samples to train the model and then evaluated its performance by calculating AUC and other metrics such as *F*1 score, mean absolute error (MAE) and Spearman correlation on the remaining 20% (17/83) of the samples see section 4. This process was repeated 100 times, and the average AUC, *F*1 score and MAE were calculated to compare the performances of the models generated using different metrics and analytical methods. This comparison revealed several interesting observations, as shown in Figure 3, Figures S1–S4; Table S4.

First, we found that the four analytical methods (Elastic Net, LASSO, Decision Tree Regression, and Decision Tree Classifier) did not perform well, as evidenced by their low AUC values, low *F*1 scores, low Spearman correlation values, and high MAE values of models generated using these techniques (Figures S1–S4; Table S4). For example, the average AUC and *F*1 values never reached 0.7 and 0.5, respectively, using either DNA damage‐ or RNA‐based metrics (Figure 3, Figures S1–S4; Table S4), suggesting that not all methods are suitable for reliable age prediction using these metrics. Second, among the other six methods, only the SSB‐ and AP‐based metrics consistently achieved AUC and F1 values exceeding 0.7 and 0.5, respectively, whereas the models based on expression levels always fell short of these thresholds (Figure 3, Figures S1–S4; Table S4). These findings highlight the superior performance of DNA damage‐based metrics over gene expression in predicting age across multiple tissues. Third, the performance of the LPKM‐SSB and LPKM‐AP metrics was highly similar for each gene set and method (Figure 3, Figures S1–S4; Table S4), indicating that both metrics are promising and reliable predictors of aging across diverse tissue types. Fourth, strikingly, using all genes as inputs into the models proved ineffective for predicting age across all methods. The average AUC, F1 and Spearman correlation values obtained for all genes were significantly lower than those derived from the top 500 age‐associated genes for all three metrics and all six well‐performing methods. Specifically, the average AUC values across all six well‐performing methods based on all genes were only 0.497 and 0.547 for the SSB‐ and AP‐based metrics, respectively, compared with the corresponding values of 0.794 and 0.793 obtained using the top 500 genes (Figure 3; Table S4).

Fifth, the six methods could be classified into two tiers based on the relationship between the overall performance and the number of input genes. The performance of the Tier I methods, Logistic Regression, Random Forest Classifier, and Naive Bayes, improved as the number of input genes increased from 15 to 500, peaking at 500 genes (Figure 3, Figures S1–S4; Table S4). For example, the average AUC values based on the top 15 LPKM‐SSB‐selected genes for the three methods ranged from 0.72 to 0.79. However, when all the top 500 genes were used to build the models, these values increased significantly and ranged from 0.87 to 0.99, as shown in Table 1 for the top‐performing Logistic Regression models. In contrast to the Tier I methods, the performance of the Tier II methods (LGBM Classifier, XGBoost Classifier, and AdaBoost Classifier) increased initially and then decreased as the number of input genes increased (Figure 3, Figures S1–S4; Table S4). For example, the peak AUC was achieved using the AdaBoost Classifier with the top 25 genes selected using the LPKM‐SSB metric and the 50 genes selected using LPKM‐AP (Figure 3; Table S4). Importantly, the Tier I methods showed superior performance, with the average AUC values ranging from 0.8 to 1 and *F*1 scores ranging from 0.7 to 1, while those for the Tier II methods ranged from 0.6 to 0.8 and 0.5 to 0.7, respectively (Figure 3, Figures S1–S4; Table S4). The lists of the top genes for each metric and their importance in the top‐performing Logistic Regression models are provided in Table S5.

**TABLE 1:** Performance of the age prediction models based on each of the three metrics.

	LPKM‐SSB	LPKM‐AP	TPM
AUC	0.99	0.99	0.64
*F*1 score	0.98	0.98	0.43
Spearman correlation	0.97	0.94	0.41
MAE	0.04	0.06	1.01

*Note*: For each metric, models were generated using logistic regression, and the top 500 genes were used.

Both LPKM‐SSB and LPKM‐AP outperformed the TPM metric in age prediction, indicating a potential association between the level of DNA damage in specific genes and aging. To determine whether this association could be partially explained by the expression levels of genes involved in the DNA damage response (DDR) pathway, we generated age prediction models using Logistic Regression and the TPM values of 210 DDR genes expressed in the six tissues (Table S6) for the same samples. This process was repeated 100 times. The age prediction models based on the DDR genes had average AUC and F1 values below 0.6 and 0.3, respectively, which were significantly less than the corresponding values of the models generated using the top 200 genes (Figure S5; Table S6). However, compared with the randomly selected 210 expressed genes, the DDR genes exhibited significantly improved performance (Figure S5; Table S6). Thus, the expression levels of DDR genes do associate with age and/or this association is less influenced by the tissue type than that of randomly‐selected genes. This association therefore could likely contribute to the age prediction ability of the two DNA damage‐based metrics. However, the relatively low age prediction ability of the DDR genes suggests that additional factors are involved in the observed association between the levels of DNA damage in specific genes and age (see Discussion section).

Finally, we repeated the analysis by calculating the LPKM‐SSB and LPKM‐AP metrics for each gene by including SSBs and AP site mapping within the entire gene boundaries (exons + introns) instead of limiting the locations of the DNA lesions to exons. As shown in Figure S6 (Tables S7 and S8), the performance patterns of different analytical methods using whole gene‐based metrics were similar to those obtained using exons only. In summary, our analysis, encompassing 10 different machine‐learning methods, varying numbers of input genes (ranging from 15 to 500), and distinct genomic features (exons or genes), showed the effectiveness of LPKM‐SSB and LPKM‐AP as robust metrics for age prediction and their superior accuracy and precision compared with RNA‐based metrics (see Discussion section).

### 3.3 DNA damage‐based biomarkers can differentiate other biological states

**FIGURE 4:**
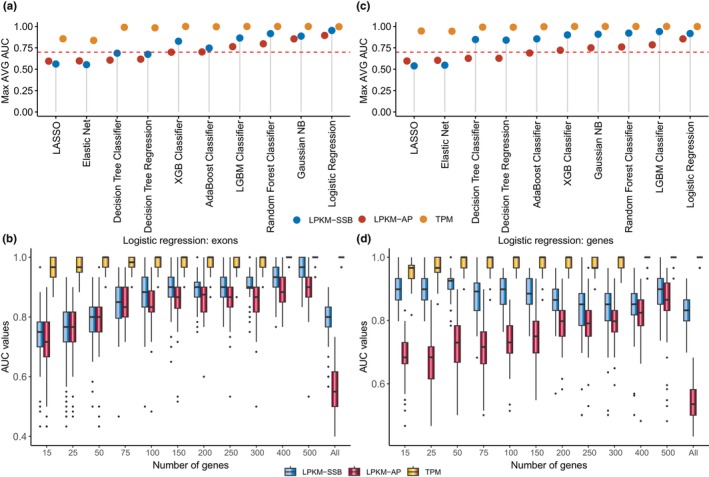
Performance evaluation of the tissue type predicting models generated by the different analytical methods. (a, c) The maximum AUC values (*Y*‐axes) achieved for each method and metric. (b, d) Boxplots of the AUC values (*Y*‐axes) of the 100 iterations for each gene set (*X*‐axes) and metric found for the top‐performing method—Logistic Regression. Box plots indicate median (middle line), 25th, 75th percentile (box), and 1.5× interquartile range (whiskers). The DNA damage metrics were calculated using either the exons (a, b) of each gene or whole genes including exons and introns (c, d). The TPM metrics were always calculated using exons only.

The surprisingly powerful performance of DNA damage‐based metrics in predicting age prompted us to investigate whether they could also be used to identify biomarkers of other biological states. To investigate this, we chose tissue type as an example of a biological state that differs with age. Similar to the age prediction analysis, we selected the top 500 tissue‐associated genes across the six tissues using ANOVA based on each of the three metrics. Likewise, we employed the same 10 machine‐learning methods to construct models for tissue‐type classification (Figure 4, Figure S7; Tables S9 and S10) and arrived at the following conclusions. First, the models based on RNA‐based metrics exhibited excellent performance in identifying tissue types (Figure 4, Figure S7; Table S10). All 10 analytical methods proved effective in tissue‐type prediction (with average AUC values >0.7), with eight achieving AUC values as high as 1 using the top 500 genes (Figure 4, Figure S7; Table S10). Interestingly, we observed that even when considering expression levels of all genes, successful tissue separation could still be achieved with AUC values of 1 using 8 of 10 methods, with only Elastic Net and LASSO failing this task (Figure 4, Table S10). This outcome differed significantly from age prediction, in which the use of all genes as inputs failed to generate adequate aging models.

Second, the RNA‐based metric outperformed the SSB‐ and AP‐based metrics in tissue‐type classification, and the TPM‐based models consistently demonstrated significantly higher AUC values across all 10 analytic methods with various gene set sizes. Third, the SSB‐ and AP‐based metrics still exhibited potential for predicting tissue types using specific machine‐learning methods. For instance, in Logistic Regression models, the average AUC values for the top 500 genes were as high as 0.95 and 0.89 using LPKM‐SSB and LPKM‐AP, respectively, while the TPM‐based model achieved an AUC value of 1. Thus, although the models based on DNA damage‐based metrics showed somewhat lower performance than those based on gene expression, they were still adequate for detecting other biological states.

Fourth, unlike the age predictions, the performances of the two DNA damage‐based metrics were similar; the models based on the LPKM‐SSB metric outperformed those based on LPKM‐AP in the tissue‐type classification task (*p* < 2.16E−16, Two‐tailed Student's *t* test). As depicted in Figure 4, eight of the 10 machine‐learning methods achieved higher AUC values for LPKM‐SSB than for LPKM‐AP. Finally, similar to the age prediction analysis, we obtained similar tissue prediction outcomes using DNA damage metrics calculated using exons only or whole genes (Figure S8; Tables S11 and S12). These findings suggested that DNA damage‐based metrics, particularly SSB‐based metrics, can serve as informative biomarkers for biological states other than age.

### 3.4 DNA damage‐based metrics reveal different subsets of biomarkers

**FIGURE 5:**
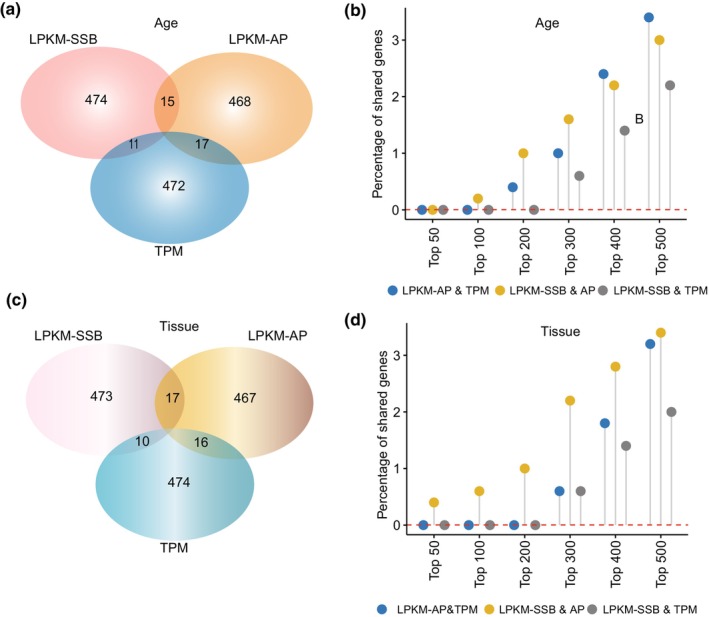
DNA damage‐based metrics identify unique sets of biomarkers. (a, c) Venn diagrams illustrating the overlap among the top 500 age‐ or tissue‐associated genes as determined by the ANOVA using the three different metrics. (b, d) The percentage of shared genes (*Y*‐axis) in the various strata of top age‐ or tissue‐associated genes (*X*‐axis) as determined by the ANOVA using the three different metrics.

Strikingly, we found little overlap among the three sets of the top 500 age‐related genes identified using these three metrics (Figure 5a,b; Table S13). First, no genes were common to any of the three sets. Second, despite 11–17 genes being shared in pairwise overlaps, none of these overlaps were statistically significant (Figure 5a; Table S13). Third, we found no increase in the significance of the pairwise overlap upon increasing the strictness of the association with age, in fact the overlap decreased by selecting genes with increasing age association (Figure 5b; Table S13). For example, there was no pairwise overlap among the top 50 age‐related genes (Figure 5b; Table S13). Fourth, the Gene ontology (GO) analysis revealed significant enrichment of different functional terms in the three 500 age‐related gene sets (Tables S14–S16). We used the GOSemSim software package (Yu et al., 2010), which formally compares two GO term profiles on a scale of 0–1 to estimate the functional similarities between the three profiles. The values were relatively low and ranged from 0.21 to 0.34 (Table S17).

Similar to the age‐related genes, different metrics revealed different subsets of tissue‐specific genes. No genes were common to all three top 500 tissue‐related genes identified by the three metrics, and a few genes were shared pairwise but without statistical significance (Figure 5c,d). Likewise, GO analysis revealed different functional categories among the three sets of 500 genes (Tables S18–S20). These results show that the different DNA damage‐based metrics revealed different age‐ or tissue‐associated genes, which also differed from those revealed by transcriptome profiling (see Section 3).

**FIGURE 6:**
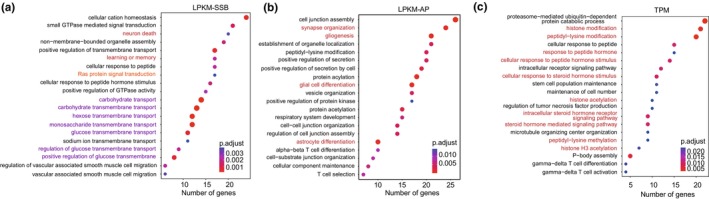
The GO enrichment results for the top 500 genes. The top 20 GO terms enriched in the top 500 age‐associated genes identified using the LPKM‐SSB (a), LPKM‐AP (b), or TPM (c) metrics.

GO analysis revealed the enrichment of functions related to the central nervous system in both the top LPKM‐SSB and LPKM‐AP age‐related gene sets (Figure 6; Tables S14–S16). Specifically, LPKM‐SSB genes were enriched in “learning or memory,” “neuron death,” and “cognition” GO terms (Figure 6a; Table S14), while the LPKM‐AP genes were enriched in “gliogenesis,” “glial cell differentiation,” “astrocyte differentiation,” and “synapse organization” (Figure 6b; Table S15). These results are consistent with previous reports linking defects in the components of SSB and base excision repair (BER) systems to neurodegeneration (Akbari et al., 2015; Caldecott, 2008; Rulten & Caldecott, 2013). However, these observations also open up a new angle that associates DNA damage with specific genes involved in the central nervous system function and aging. In addition, GO analysis revealed multiple enriched terms related to hexose transport and Ras protein signaling in the LPKM‐SSB gene set (Table S14). Alteration of sugar metabolism is a hallmark of aging (Amorim et al., 2022; Chia et al., 2018), and Ras signal transduction is an important modulator of this process (Slack, 2017), which is potentially consistent with the associations between the levels of DNA damage in these genes and age. In contrast, the age‐related gene set identified through TPM values exhibited a significant enrichment of functions related to histone modifications and hormonal signaling (Figure 6c; Table S16).

## 4 DISCUSSION

In this study, we found that the levels of DNA damage in certain genes determined based on genome‐wide profiles of SSBs and AP sites offer a new and promising source of age‐related biomarkers based on two lines of evidence. First, we detected a higher fraction of genes significantly correlated with age using DNA damage‐based metrics than RNA‐based metrics across the six different tissue types. This analysis did not involve any preconceived feature selection process, suggesting that the DNA damage‐based of biomarkers are less influenced by tissue type than RNA‐based biomarkers. Second, age prediction models based on the analysis of genome‐wide patterns of DNA damage have higher precision and accuracy than those achieved using transcriptome analysis (also see below), which has been widely used as an age prediction biomarker (Bao et al., 2023). This conclusion was reached using different machine‐learning approaches, arguing that it was not an artifact of a specific analytical procedure. Moreover, the performance of the RNA‐based metric was superior to that of the DNA damage‐based metric in predicting other biological states, such as tissue type, when tested using the same array of machine‐learning methods. If the conclusions based on these methods were skewed to benefit DNA damage‐based metrics, we would expect the same to occur in both age‐ and tissue‐type prediction analyses; however, this was not the case.

In our previous genome‐wide analyses of the patterns of SSBs and AP sites, we found that their distribution was not random (Cai et al., 2022, Cao et al., 2019, 2022). DNA lesions exhibit complex yet dynamic genomic patterns that depend heavily on the tissue type (Cai et al., 2022; Cao et al., 2019). These results are consistent with a large body of previous work that has shown that the generation and repair of DNA lesions are not random in a genome but are influenced by multiple factors, such as transcription, chromatin state, and local sequence composition (Bohr et al., 1987; Frigola et al., 2017; Perera et al., 2016; Sassa & Odagiri, 2020; Ye et al., 1998). Moreover, DNA repair efficiency decreases with age (Chen et al., 2020; Maynard et al., 2015). Therefore, it is not surprising that genomic patterns of DNA damage can be used as biomarkers for aging.

However, the conclusions of this study differ somewhat from those obtained in our previous study, in which we analyzed the same data and failed to find age‐related biomarkers based on DNA damage that were consistent across different tissues (Cai et al., 2022). We believe that the main reason for this is that in the current study, we first identified the top‐performing features, the top 500 age‐related genes, and then used these features as the input into various machine‐learning methods. In our previous study, we used all genomic features of a particular type to identify associations with age without prior selection (Cai et al., 2022). However, as shown in the current study, all genes performed poorly as age predictors for any DNA damage‐ or RNA‐based metric. One possible reason for this is that incorporating all features might result in nonspecific noise that is not related to age and, therefore, adversely affect the performance of the models. It is also important to mention that different machine‐learning methods show very different performance levels in age prediction using any of the three metrics. Thus, selecting an appropriate analysis method and input features also plays a vital role in accurate age prediction.

Our results indicate the existence of a (relatively) small set of genes that exhibit consistent age‐related changes in the total load of DNA lesions across different tissues. These genes have functions related to the central nervous system, sugar metabolism, and Ras signaling. The age‐related genes identified by measuring changes in SSBs and AP sites differ and have little overlap with each other, as well as with genes identified using RNA profiling. However, considering that each of the three metrics (LPKM‐SSB, LPKM‐AP, and TPM) used in this study measures different events, the uniqueness of the corresponding gene sets is not unexpected. Although both the SSBs and AP sites represent DNA damage events, their modes of generation differ. For example, some of the mechanisms that could lead to detectable SSBs in this study, such as topoisomerase activity or stalling of replication forks (among others), would not be expected to generate AP sites. Similarly, although we found a significant relationship between the expression level and the overall load of SSBs and AP sites, which is consistent with multiple studies that reported a relationship between transcription and DNA damage, the association was relatively modest (Cai et al., 2022). Thus, differences in transcription patterns and the two types of DNA lesions would be expected.

Currently, we do not know if DNA damage accumulation in all or some of these genes is directly related to aging or if it represents an indicator of aging or some aging‐related processes, for example, changes in DNA repair efficiency. However, we found that DNA damage levels were negatively correlated with age for some genes. At face value, our results appear to contradict multiple reports documenting an overall decline in DNA repair with age and, more generally, a strong causal relationship between DNA damage and aging (Campisi, 2013; Chen et al., 2020; Hoeijmakers, 2009; Maynard et al., 2015; Ou & Schumacher, 2018; Robert & Wagner, 2020; Yousefzadeh et al., 2021). However, the following interpretative caveats must be considered. First, excessive unrepaired DNA damage can lead to cell death or cellular senescence, as defined by irreversible replication stoppage (Campisi, 2013; Ou & Schumacher, 2018). On the other hand, in this work the DNA damage was measured in tissues or cellular populations that are expected to mostly consist of live cells that likely have relatively low levels of DNA damage. Thus, the age‐related increase in DNA damage that causes cell death or senescence would likely have a relatively small contribution to the overall signal detected here. Second and related to the first point, one of the negative effects of DNA damage is the accumulation of somatic mutations with age caused by improper DNA repair (Cagan et al., 2022). However, in this work, we measured only the steady‐state levels of SSB and AP sites and not the outcome of the repair of these lesions.

Third, a growing number of reports have shown that, in addition to its detrimental role, DNA damage, including SSBs and AP sites, can have physiologically important roles in the cell, for example, in regulating gene expression (Bunch et al., 2015; Ju et al., 2006; Larsen et al., 2010; Le May et al., 2012; Madabhushi et al., 2015; Perillo et al., 2008; Puc et al., 2015; Puc et al., 2017; Trotter et al., 2015). Therefore, the decrease in the levels of these lesions in certain genes may be related to mechanisms regulating the expression of these genes.

Finally, the DNA lesions measured in this study, SSBs and AP sites, represent both the original DNA lesions and intermediates of DNA repair. For example, multiple types of DNA base damage are repaired by BER, which first converts these lesions to AP sites and then to SSBs (Dianov & Hubscher, 2013). Thus, the decrease in SSBs or AP sites in certain genes with age might reflect a decrease in the activities of BER and other types of DNA repair pathways. Altogether, the steady‐state levels of DNA damage detected in this study likely reflect a complex interplay between the rates of DNA damage generation and repair in live cells, which vary across the genome and can be influenced by age and tissue/cell type. In fact, a complex relationship between various types of DNA damage, including AP sites, and age were previously reported by other groups (Flasz et al., 2022; Soares et al., 2014).

In this study, we did not compare DNA damage‐based biomarkers with all other types of age biomarkers, such as those based on various epigenomic features (e.g., DNA methylation profiles) and proteomic analysis. However, it is important to emphasize that we achieved AUC values of approximately 1 in some age prediction models based on the DNA damage‐based metrics. Therefore, even without these comparisons, our results show that genome‐wide DNA damage profiles can provide age biomarkers, which would be no worse than the other best predictors of age. Furthermore, DNA damage‐based metrics can differentiate other biological states with performance levels comparable to those of other common biomarkers. Furthermore, from a practical point of view, the overall cost of an SSB survey, including library preparation and next‐generation sequencing, can be as low as 10–20 USD, which is 1–2 orders of magnitude lower than the cost of transcriptome profiling using RNA‐seq or total methylome analysis with bisulfite sequencing. All DNA damage surveys reported here were performed using genomic DNA directly isolated from tissues or cells using commercially available kits (Cai et al., 2022; Cao et al., 2022), without the crosslinking or fragmentation steps used in the original version of the SSiNGLe protocol (Cao et al., 2019).

However, while the transcriptome‐based age prediction models generated in this work underperformed compared with those based on DNA damage, this does not necessarily mean that better models based on gene expression cannot be developed. In this study, we used RNA‐seq based on bulk sequencing to benchmark the performance of the DNA damage‐based metrics which currently can only be obtained by bulk sequencing. However, several promising age prediction models based on single‐cell RNA‐seq have been recently developed (Buckley et al., 2023; Holzscheck et al., 2021). Furthermore, it is possible that different feature selection approaches or modeling algorithms can improve the performance of age prediction models based on bulk RNA‐seq.

Overall, this study shows for the first time that DNA damage‐based metrics derived from high‐resolution whole‐genome maps of DNA damage have strong potential as biomarkers of age. Furthermore, it shows that the relationship between the level of DNA damage in normal tissues and age is complex, with some genes showing a decline in the total DNA damage in older animals. DNA damage‐based metrics have the following potential advantages: (1) low cost, particularly in the case of LPKM‐SSB, (2) less influence by tissue type, and (3) ability to uncover novel biological phenomena associated with aging.

However, this study was based on a limited number of animals (2–3 per time point and tissue type) of a single species and sex. Thus, further similar studies with much larger sample sizes are required to assess the validity of these conclusions in other mammals, especially humans, and across sexes. Additional studies are required to fully understand the performance of DNA damage‐based metrics relative to other biomarkers. Nonetheless, the current study highlights that the genomics of DNA damage represents a very young and unexplored area with a wealth of useful information that can significantly enrich practical applications and basic science.

## AUTHOR CONTRIBUTIONS

PK and HC conceived the project. PK and HC wrote the manuscript. HC and PK supervised the project. DB and HC performed computational analyses with the help of YC, FY, TS, YZ, and CW. HC wrote the first draft of the manuscript, with contributions from JL, LX, XC, and YD.

## FUNDING INFORMATION

No funding information provided.

## CONFLICT OF INTEREST STATEMENT

The authors declare that this study was conducted in the absence of any commercial or financial relationships that could be construed as potential conflicts of interest.

## Supporting information


Appendix S1.

Appendix S2.

## Data Availability

The next‐generation sequencing data, the coordinates of the SSB and AP sites, and the expression levels (TPM values) of genes generated in this study have been deposited in the GEO database under accession codes GSE190955 and GSE239751 (data from the 24‐month‐old animals generated in this study). The processed data generated in this study are provided in Supplementary files. The custom code used to generate the coordinates of SSBs and AP sites is available on GitHub (https://github.com/huifencao/DB‐Nature_Communications‐2019) and Zenodo (10. 5281/zenodo.7013389). The code used to construct the machine‐learning models for predicting age or tissue type is available on GitHub (https://github.com/dapao111/DNA_damages_parallel_analysis.git) and Zenodo (https://zenodo.org/record/8220225).
