# Supplementary material for: Genome‐wide profiles of DNA damage represent highly accurate predictors of mammalian age
Source: Aging Cell. 2024 Feb 23;23(5):e14122. doi: 10.1111/acel.14122 (PMC11113270; doi:10.1111/acel.14122)
Supplement: Supplementary file 1 — Appendix S1. [file ACEL-23-e14122-s002.pdf]

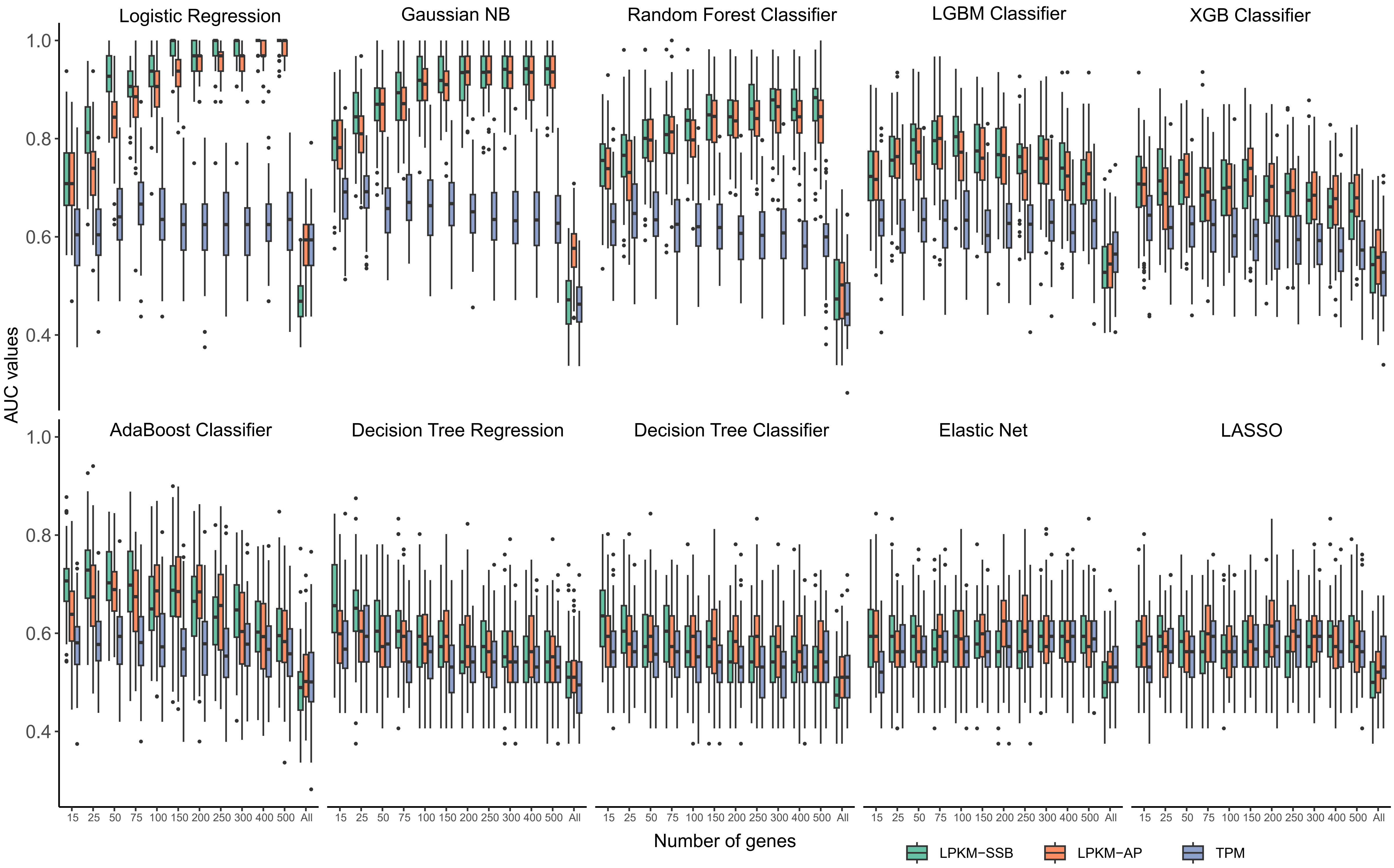

Supplementary Figure 1. Distribution of the AUC values for age prediction models obtained using the three metrics and DNA damage signal calculated based on exons. Boxplots of the AUC values (Y-axes) of the 100 iterations for each gene set (X-axes) and each metric for each method.

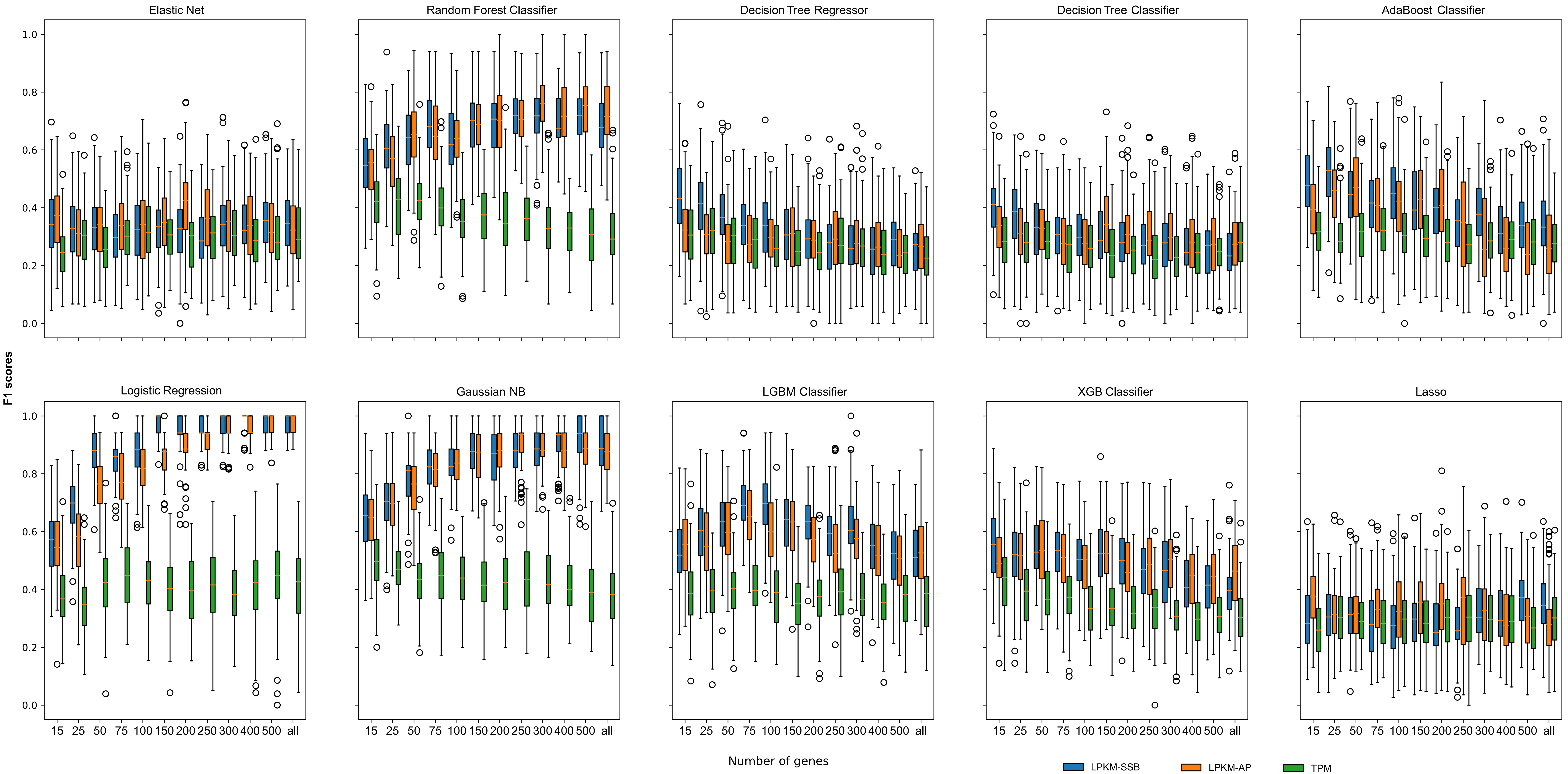

Supplementary Figure 2. Distribution of the F1 values for age prediction models obtained using the three metrics and DNA damage signal calculated based on exons. Boxplots of the F1 values (Y-axes) of the 100 iterations for each gene set (X-axes) and each metric for each method.

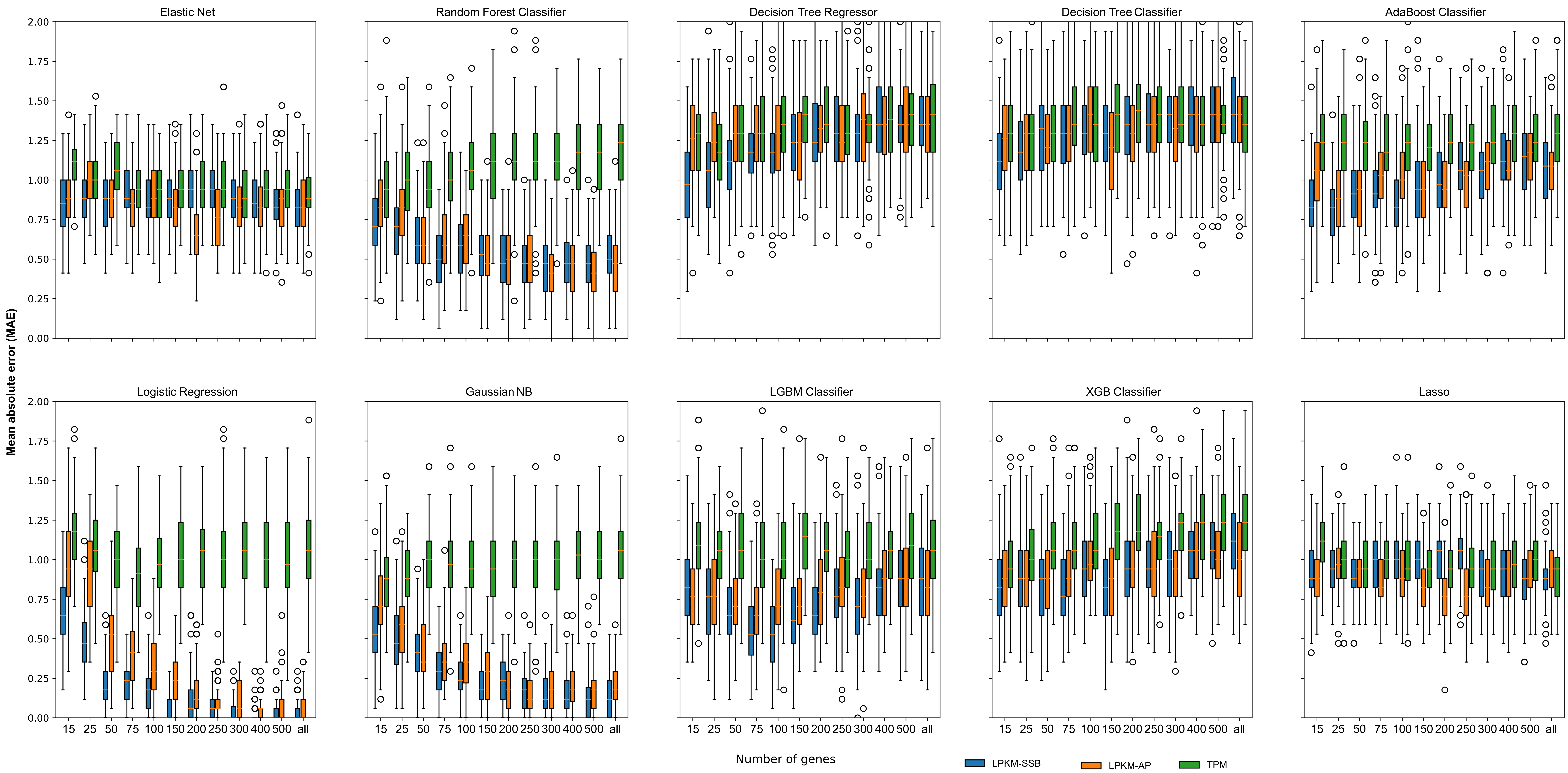

Supplementary Figure 3. Distribution of the mean absolute error (MAE) values for age prediction models obtained using the three metrics and DNA damage signal calculated based on exons. Boxplots of the MAE values (Y-axes) of the 100 iterations for each gene set (X-axes) and each metric for each method.

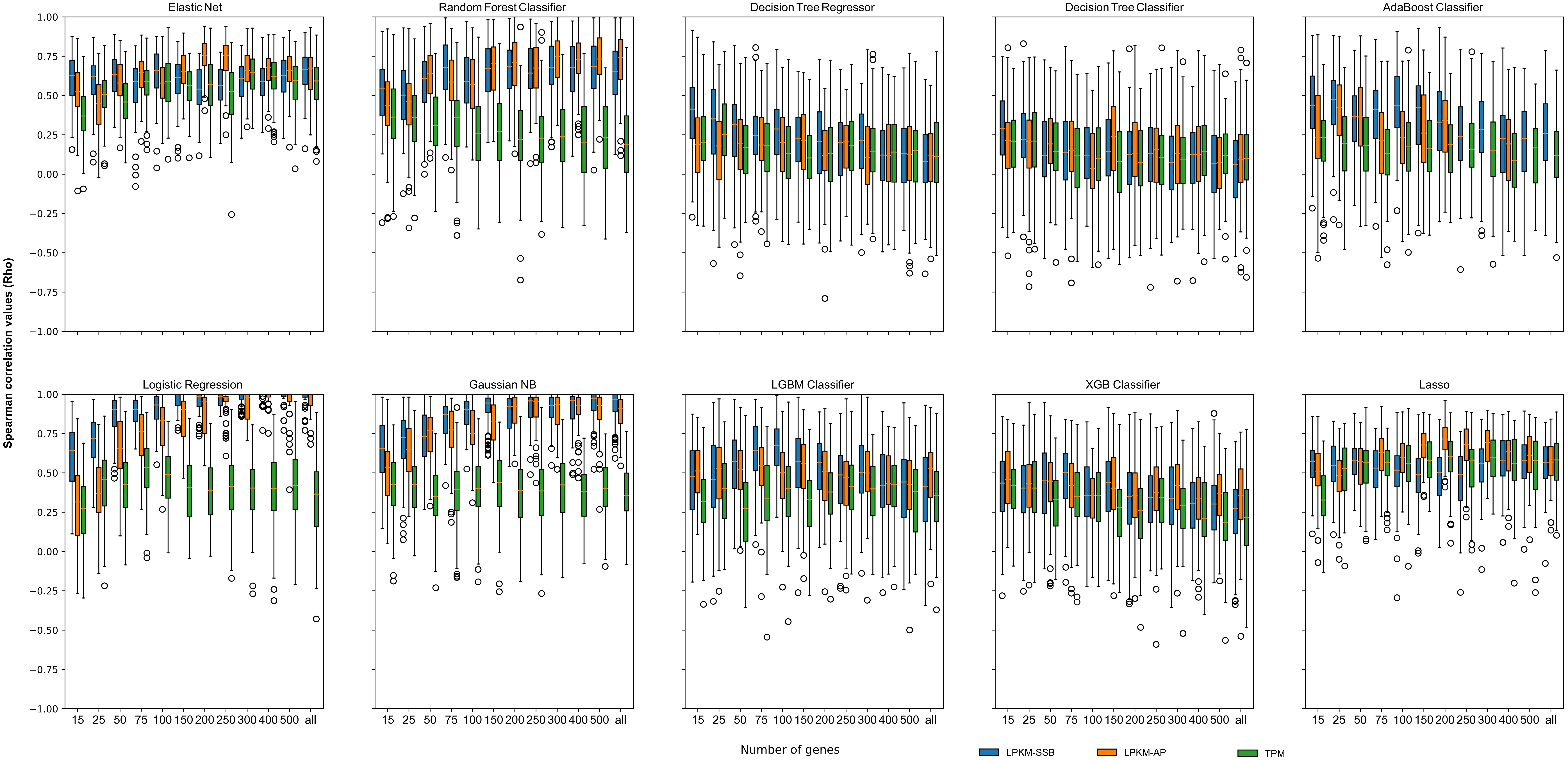

Supplementary Figure 4. Distribution of the Spearman correlation values for age prediction models obtained using the three metrics and DNA damage signal calculated based on exons. Boxplots of the Spearman correlation values (Y-axes) of the 100 iterations for each gene set (X-axes) and each metric for each method.

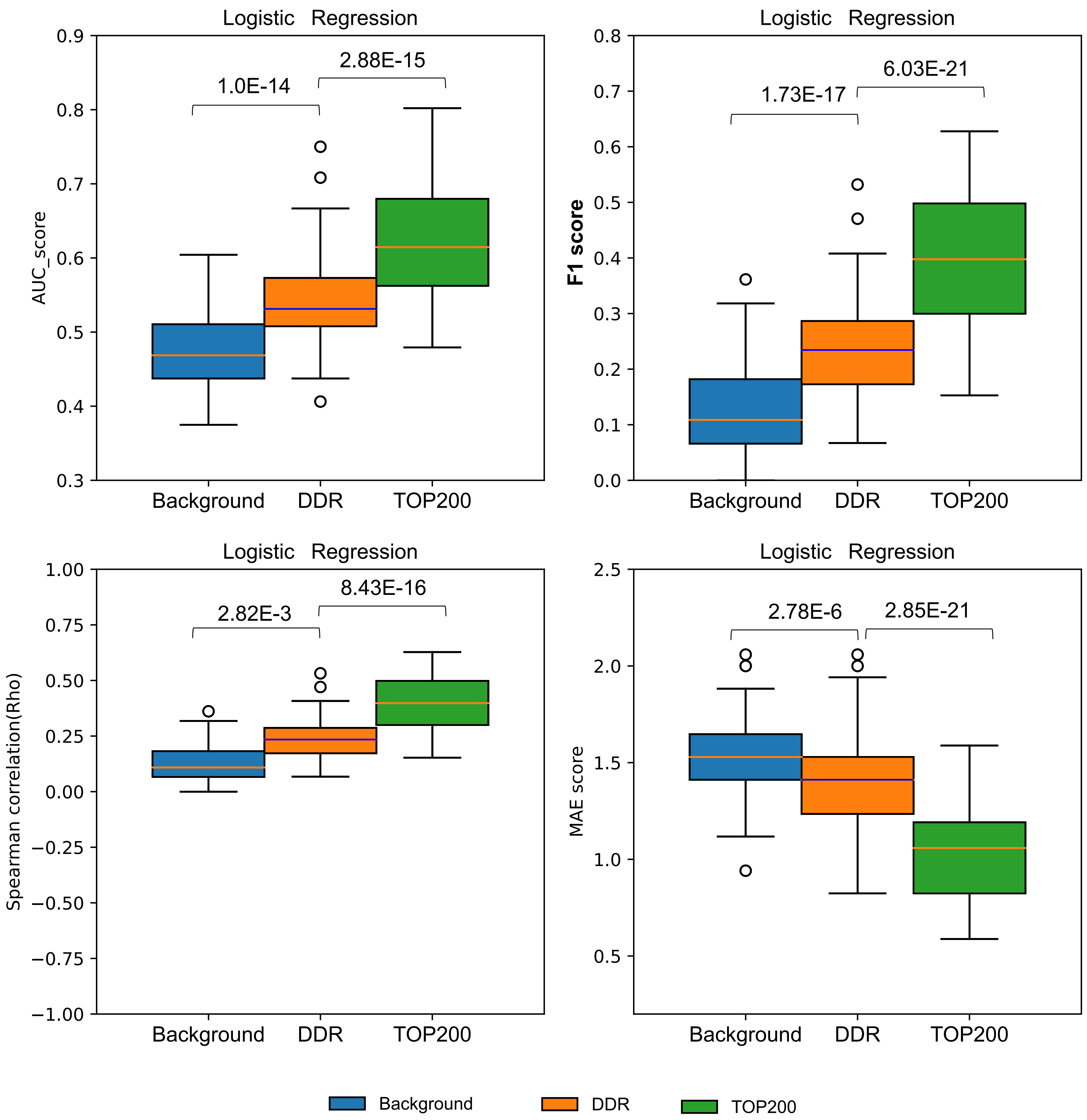

Supplementary Figure 5. Distribution of the AUC, F1, Spearman correlation and MAE values for age prediction models obtained using TPM values of 210 DNA damage response (DDR) genes. The DDR genes are listed in the Supplementary Table 6 and they were obtained from this website: https://www.mdanderson.org/documents/Labs/Wood-Laboratory/human-dnarepair-genes.html. The “background” corresponds to randomly selected 210 genes expressed in at least one tissue. “TOP200” represent the top 200 out of the top 500 genes that were selected based on the F-score analysis of the TPM values of all genes across the age groups. Boxplots show the distribution of the AUC, F1, Spearman correlations and MAE scores (Y-axes) obtained in 100 iterations of the analysis for each gene set using Logistic Regression. The p-values shown above the lines were calculated using one-tailed Student's t-test.

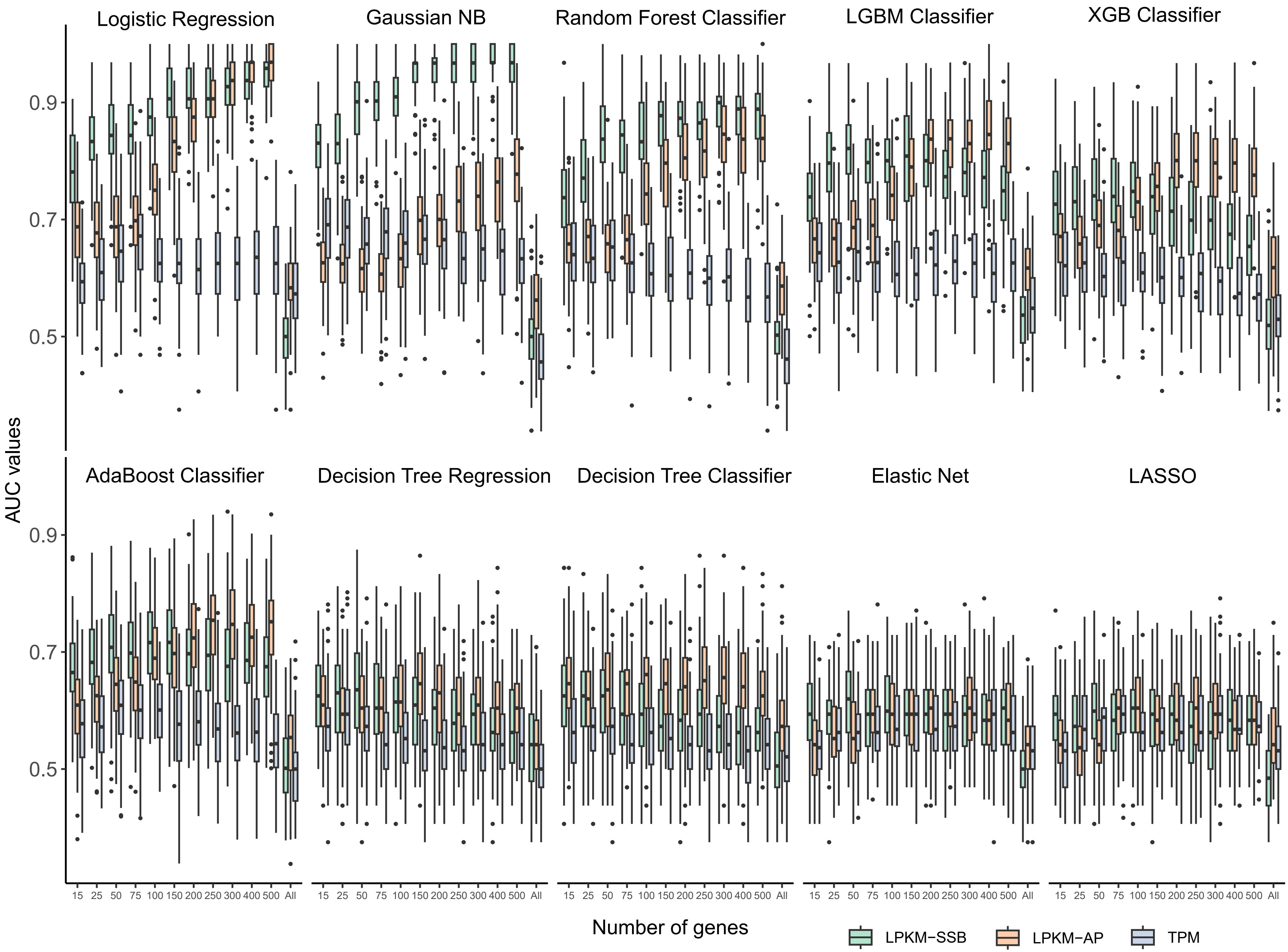

Supplementary Figure 6. Distribution of the AUC values for age prediction models obtained using the three metrics and DNA damage signal calculated based on whole genes (exons + introns). Boxplots of the AUC values (Y-axes) of the 100 iterations for each gene set (X-axes) and each metric for each method.

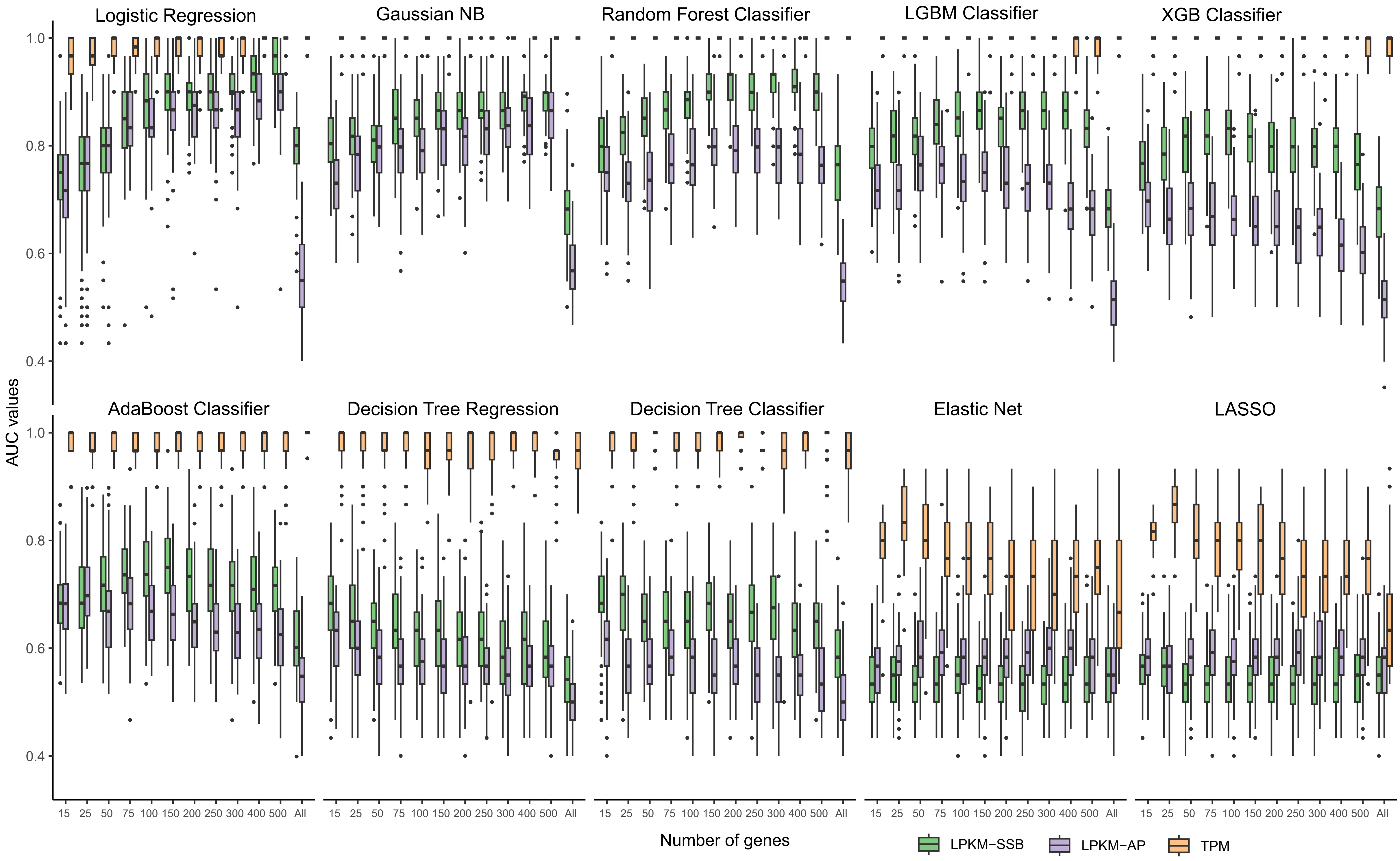

Supplementary Figure 7. Distribution of the AUC values for tissue prediction models obtained using the three metrics and DNA damage signal calculated based on exons. Boxplots of the AUC values (Y-axes) of the 100 iterations for each gene set (X-axes) and each metric for each method.

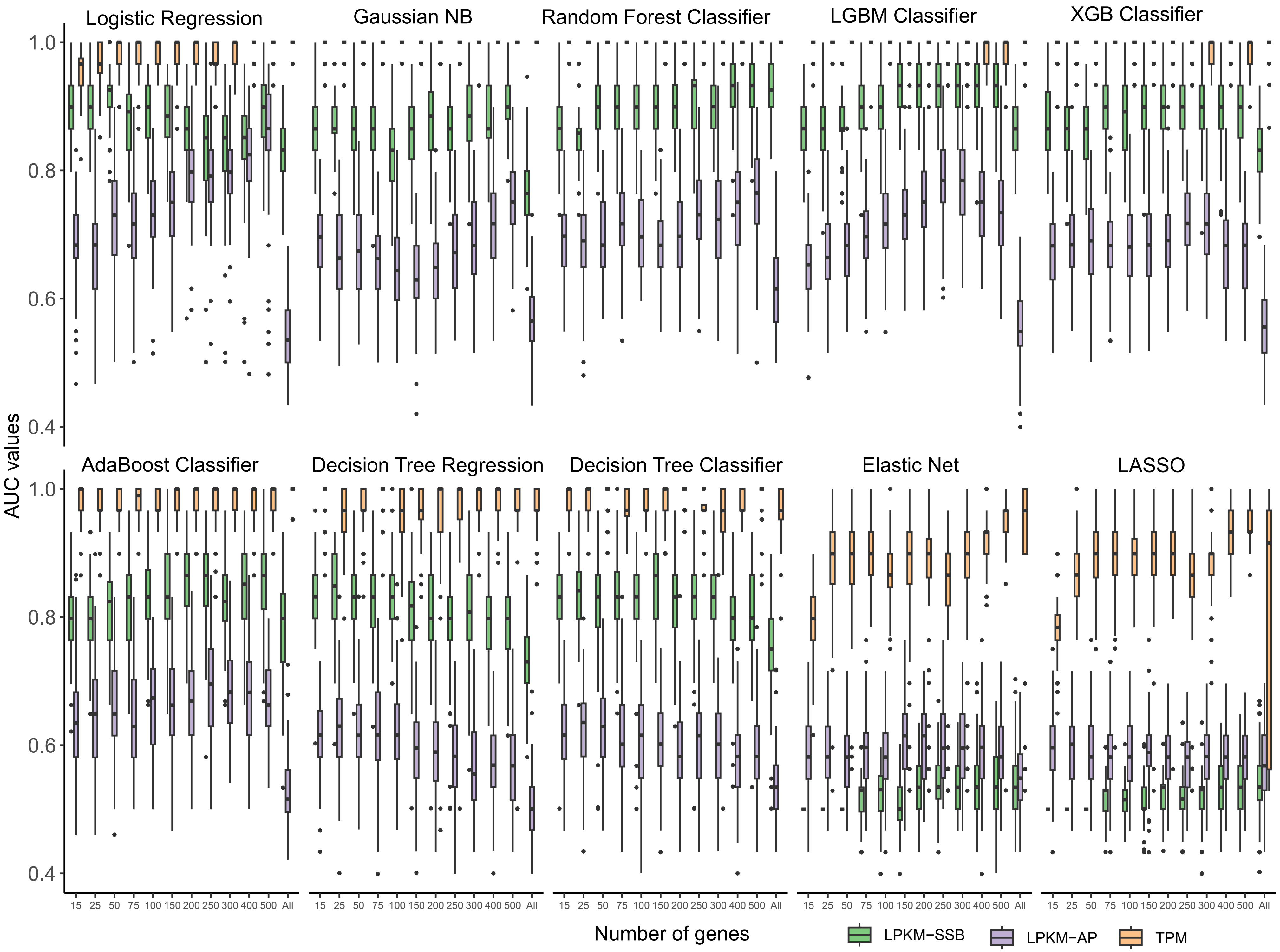

Supplementary Figure 8. Distribution of the AUC values for tissue prediction models obtained using the three metrics and DNA damage signal calculated based on whole genes (exons + introns). Boxplots of the AUC values (Y-axes) of the 100 iterations for each gene set (X-axes) and each metric for each method.
